# Severe Lactic Acidosis With Multi-organ Dysfunction Secondary to Terlipressin-Induced Widespread Vasospasm in a Patient With Liver Cirrhosis and Oesophageal Variceal Bleeding: A Case Report

**DOI:** 10.7759/cureus.103170

**Published:** 2026-02-07

**Authors:** Mst Hiramoti, Nahin Fardin Ruthi, Saqiba Batool

**Affiliations:** 1 General Internal Medicine, Luton and Dunstable Hospital, Bedfordshire Hospitals NHS Foundation Trust, Dunstable, GBR

**Keywords:** lactic acidosis, liver cirrhosis, multi-organ dysfunction, octreotide, systemic vasospasm, terlipressin, variceal haemorrhage

## Abstract

Terlipressin is widely used for acute variceal bleeding and hepato-renal syndrome, but rare complications such as severe lactic acidosis with multi-organ dysfunction due to systemic vasospasm can occur. We report a 58-year-old male with type 2 diabetes and alcoholic liver cirrhosis who developed a lactate level of 20 mmol/L and a pH of 6.97. He became systemically unwell hours after terlipressin initiation. Further investigations excluded alternative causes of lactic acidosis. Clinical improvement occurred, followed by discontinuation of terlipressin and initiation of octreotide, highlighting the need for vigilance during therapy.

## Introduction

Terlipressin is a synthetic analogue of vasopressin widely used in the management of acute gastroesophageal variceal haemorrhage and hepatorenal syndrome in patients with cirrhosis. Its therapeutic effect is mediated through V1 receptor-induced splanchnic vasoconstriction, which reduces portal venous inflow and variceal blood loss. Compared with vasopressin, terlipressin has a longer duration of action and a more favourable safety profile, resulting in fewer systemic ischaemic complications [1,2].

Despite its established clinical role, rare ischaemic adverse effects have been reported, including myocardial, peripheral, cutaneous, and mesenteric ischaemia. In exceptional cases, excessive or widespread vasoconstriction may lead to severe lactic acidosis, mimicking acute mesenteric ischaemia and posing significant diagnostic and management challenges [2].

We report a case of terlipressin-induced systemic vasospasm presenting with severe lactic acidosis and multi-organ dysfunction, which resolved following prompt drug discontinuation. This case highlights the importance of vigilance and early clinical reassessment when unexpected metabolic deterioration occurs during terlipressin therapy.

## Case presentation

A 58-year-old male presented with a one-day history of hematemesis (three episodes, ~500 mL) without melaena. His past medical history included type 2 diabetes mellitus and alcohol-related liver cirrhosis with radiological evidence of portal hypertension, which was diagnosed four months ago. He drank approximately two bottles of wine per week. As per drug history, he took metformin 1 g twice daily.

On admission, he was alert, pale, and mildly jaundiced. Vital signs were stable, with National Early Warning Score (NEWS) [3] of 1, blood pressure (BP) of 128/63 mmHg, heart rate (HR) of 105 bpm, respiratory rate (RR) of 18/min, and oxygen saturation (SpO₂) of 98% on air. Examination revealed hepatomegaly, with no other abnormalities. Per rectal (PR) examination revealed no melaena. ECG showed sinus tachycardia. Glasgow-Blatchford score (GBS) [4] was 6 (indicating high risk of bleeding). Initial venous blood gas (VBG) demonstrated metabolic acidosis with hyperlactatemia (pH = 7.32, lactate = 8.0 mmol/L). Admission full blood test showed stable liver and renal function tests (Table 1).

**Table 1:** Results of laboratory blood tests on admission. APTT: activated partial thromboplastin time; INR: international normalised ratio; NRBC: nucleated red blood cells.

Test	Result	Units	Normal range
Calcium	2.23	mmol/L	2.20–2.60
Albumin	33	g/L	35–50
Adjusted calcium	2.37	mmol/L	2.20–2.60
C-reactive protein	1	mg/L	<5.0
Prothrombin time	14.3	s	9–14
APTT	26.1	s	26–38
INR	1.2		0.8–1.2
White blood cell count	7.7	×10⁹/L	4.0–11.0
Haemoglobin	115	g/L	130–165
Platelets	121	×10⁹/L	150–450
Lymphocytes	1.52	×10⁹/L	1.0–3.0
Monocytes	0.9	×10⁹/L	0.2–1.0
Eosinophils	0.03	×10⁹/L	0.0–0.4
Basophils	0.07	×10⁹/L	0.02–0.1
NRBC	<0.5	×10⁹/L	<0.5
Mean platelet volume	9.7	fL	7.8–11.0
Total protein	68	g/L	60–80
Calculated globulin	35	g/L	—
Total bilirubin	28	µmol/L	<21
Alkaline phosphatase	85	U/L	30–130
Alanine aminotransferase	67	U/L	<41
Magnesium	0.71	mmol/L	0.70–1.00
Phosphate	1.03	mmol/L	0.80–1.50
Sodium	139	mmol/L	133–146
Potassium	4.6	mmol/L	3.5–5.3
Urea	8.1	mmol/L	2.5–7.8
Creatinine (Jaffe)	69	µmol/L	62–106
Alpha-fetoprotein	2.5	IU/mL	≤7

**Figure 1:**
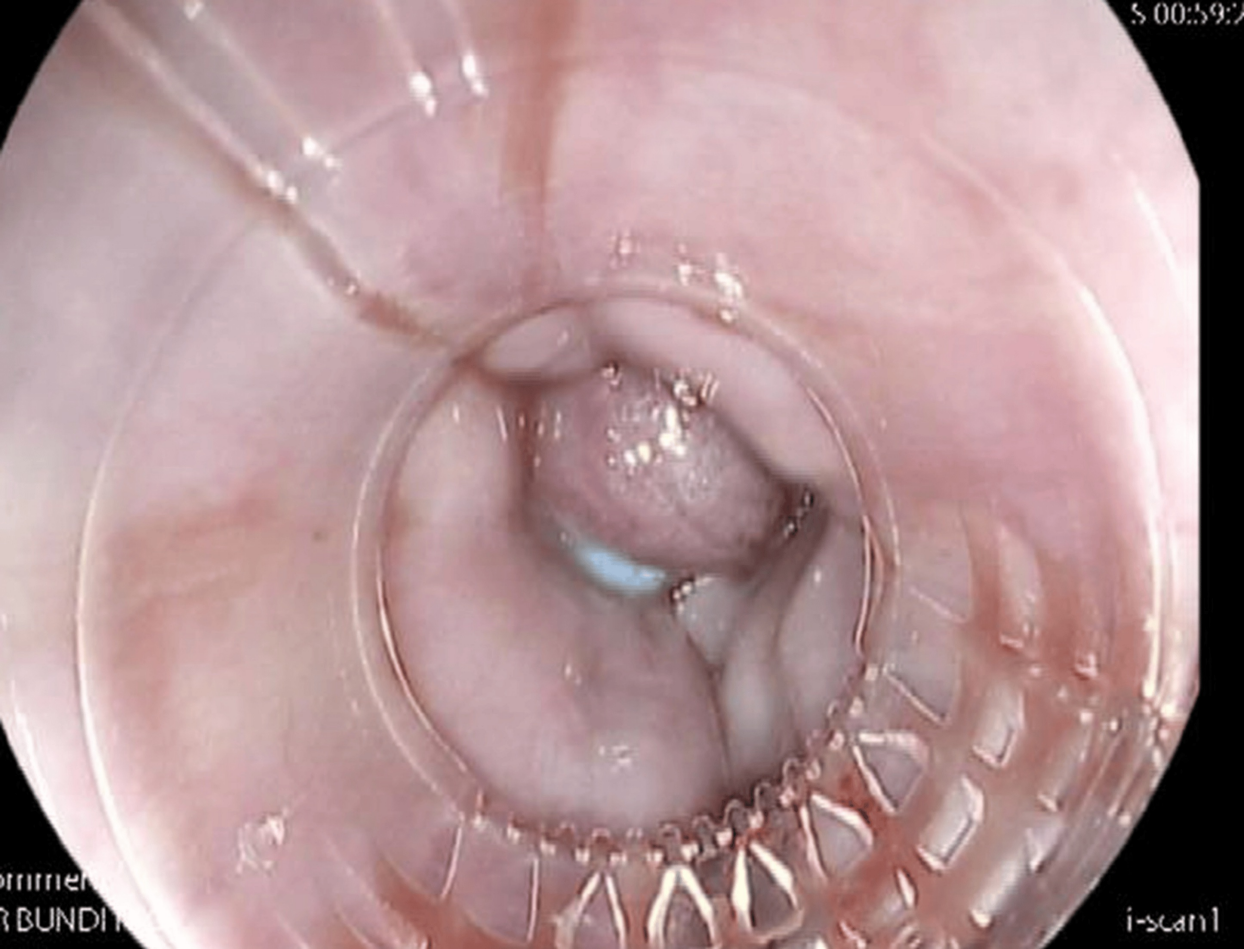
Endoscopy showed banded oesophageal varices.

Urgent endoscopy within a couple of hours of admission showed grade 3 oesophageal varices (Figure 1), treated with seven-band ligation. The management plan included initiation of terlipressin, intravenous proton pump inhibitor, antibiotics, and tranexamic acid. He was stabilised initially; however, within hours of terlipressin initiation, the patient developed acute hypotension (BP = 92/59 mmHg), tachycardia (118 bpm), drowsiness, and dizziness. VBG showed a pH level of 6.97, with a lactate level of 20 and a bicarbonate level of 6. Full blood test at the same time showed high creatinine (123 micromol/L) and high alanine aminotransferase (ALT) (312 U/L), which ultimately indicates multi-organ dysfunction in comparison to the initial blood test during admission (Table 1). The differential diagnosis included haemorrhagic shock, sepsis, mesenteric ischaemia, and drug-induced systemic vasospasm.

**Figure 2:**
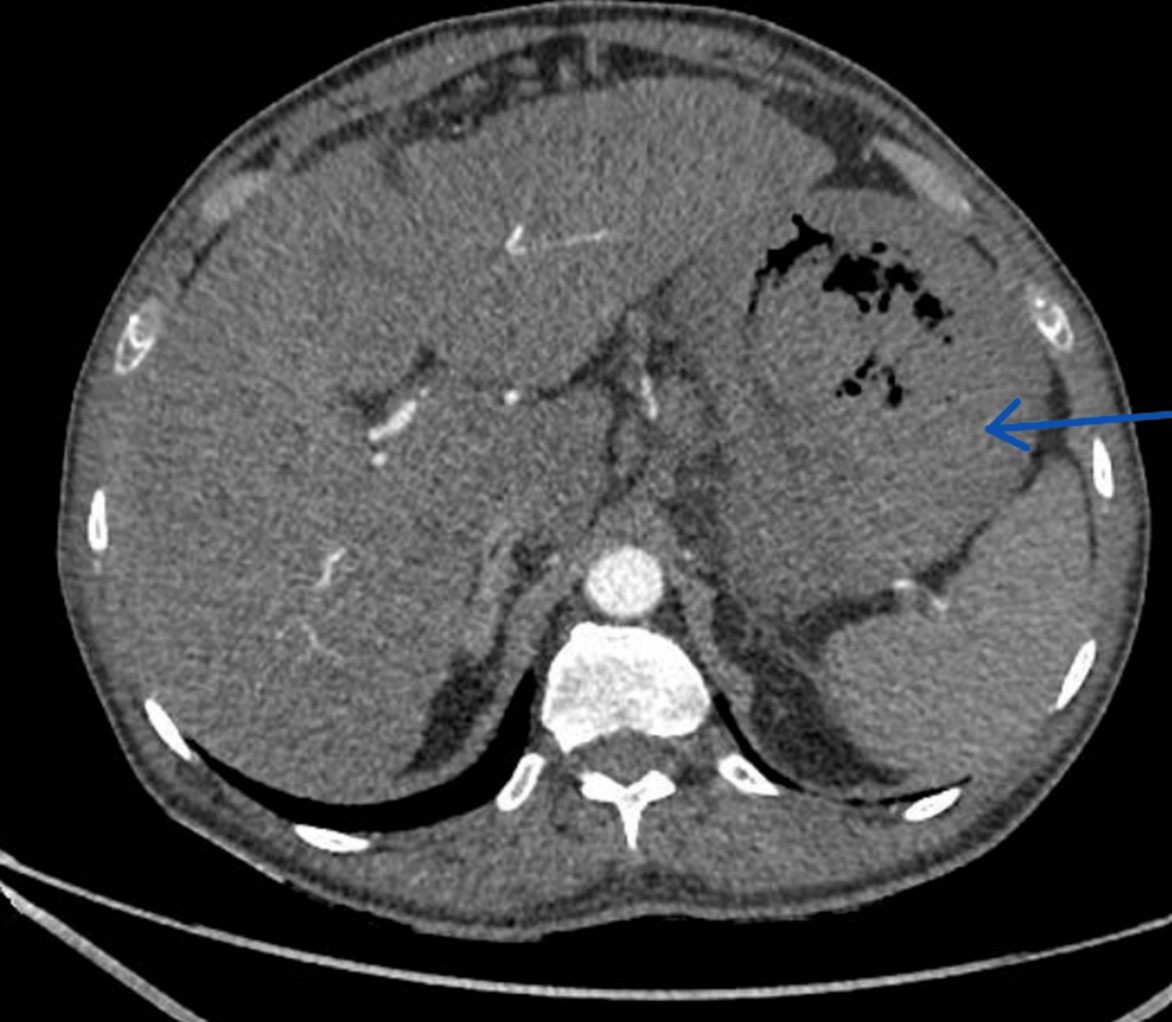
CT angiography of the abdomen. The blue arrow indicates the gastric wall is markedly thickened, and the flat planes along the lesser gastric curve show infiltration consistent with an inflammatory process or with arterial/venous ischaemia.

**Figure 3:**
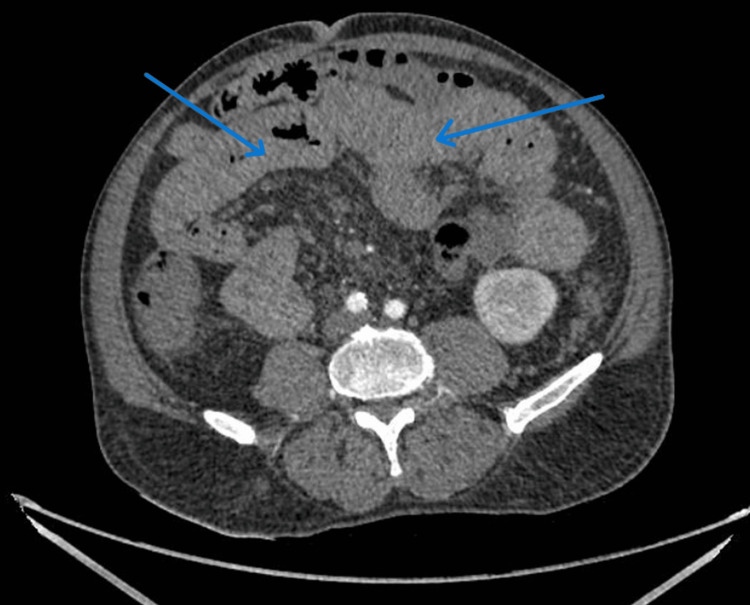
CT angiography of the abdomen. Blue arrows indicate the proximal colon and ileum, showing marked wall thickening, highly suspicious in the clinical context of arterial or venous ischaemia.

CT angiography reported patent vasculature and no obvious gut or mesenteric ischaemia, but showed a marked thickening of the wall of the stomach and colon (Figures 2, 3), likely representing temporary venous or arterial ischaemia. In view of the clinical context, it indicated that severe vascular spasm caused this bowel wall thickening. Additionally, repeat endoscopy did not show any further major upper GI bleed. The severe systemic vasospasm raised suspicion of a temporal association with terlipressin.

**Figure 4:**
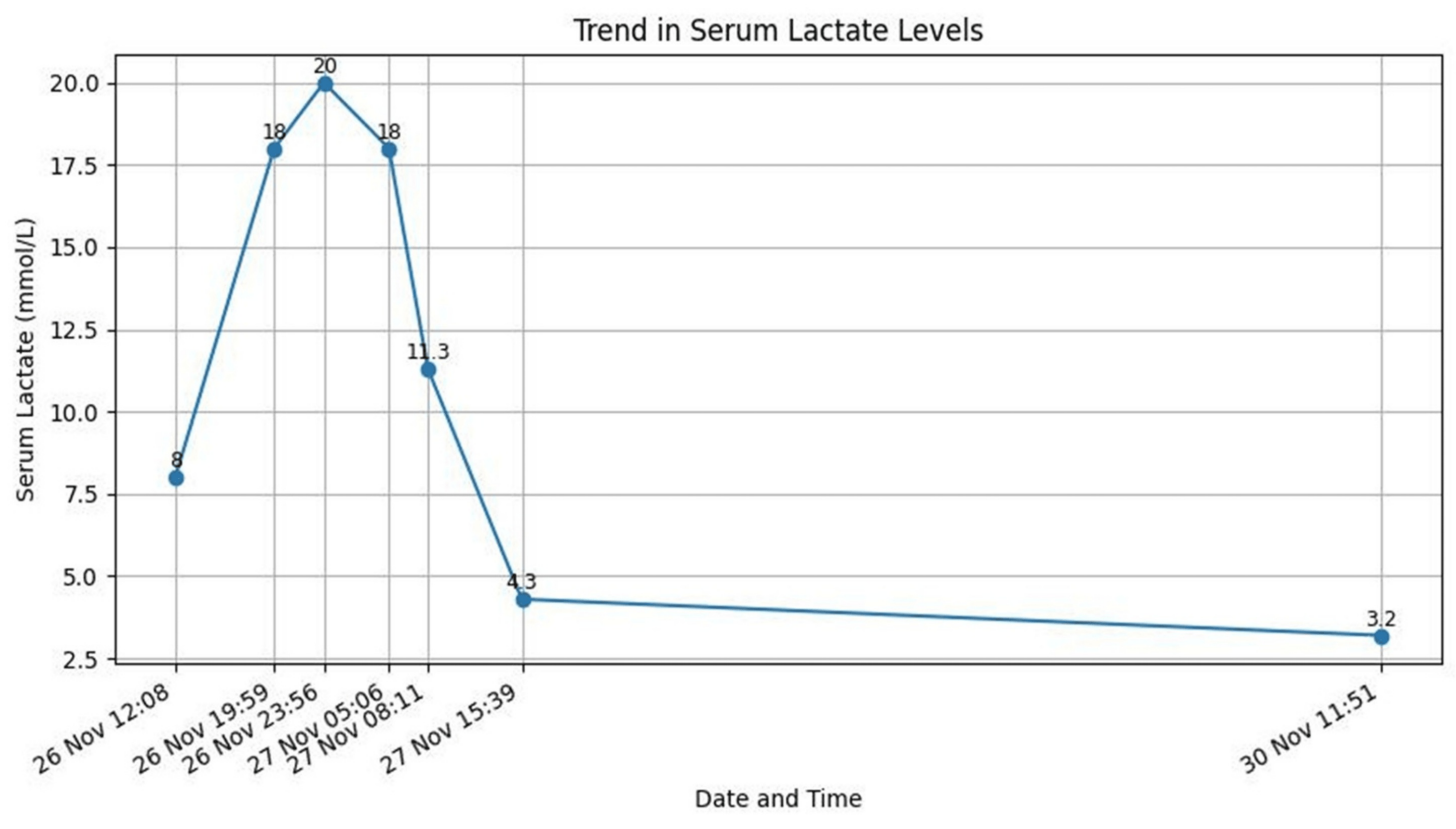
Lactate level throughout the admission.

**Figure 5:**
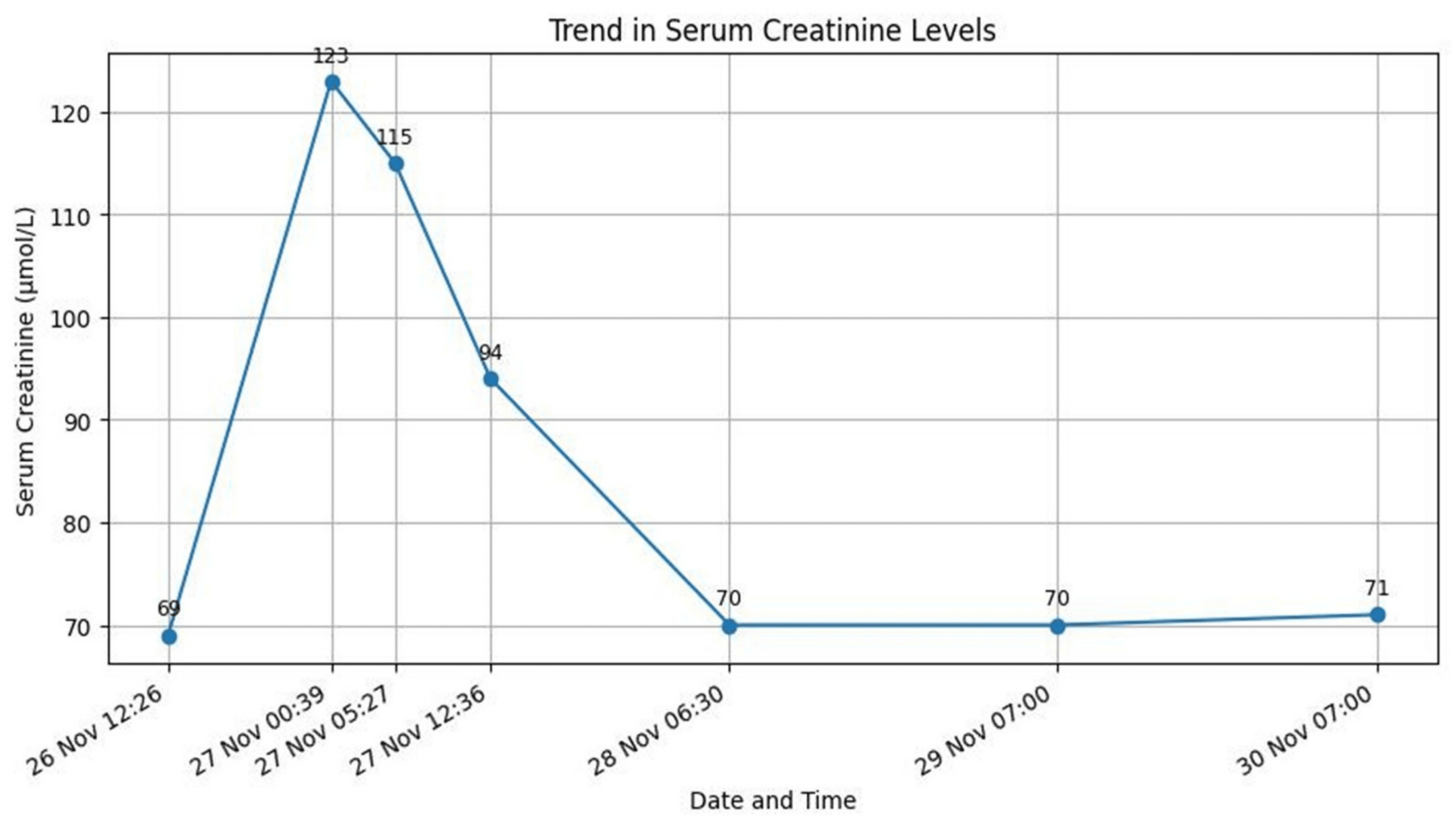
Serum creatinine level throughout the admission.

**Figure 6:**
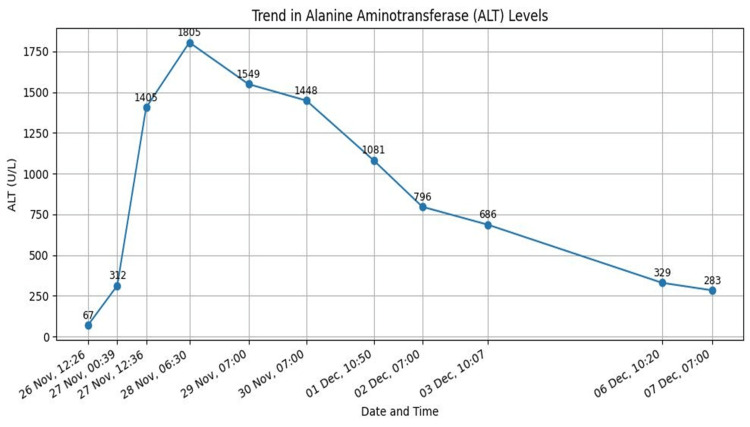
Alanine aminotransferase (ALT) level throughout the admission.

Terlipressin was discontinued, and octreotide was initiated. Lactate and acid-base status normalised within 24-48 hours (Figure 4), with no further bleeding. Blood test showed improvement in kidney and liver function tests as well (Figures 5, 6). The patient remained haemodynamically stable and was discharged with advice to avoid terlipressin permanently. Follow-up confirmed continued clinical stability.

To assess the relationship between terlipressin use and the patient’s symptoms, the Naranjo Adverse Drug Reaction Probability Algorithm was applied [5]. This validated questionnaire determines the likelihood that an adverse drug reaction (ADR) is attributable to a specific drug rather than alternative factors, categorising reactions as definite, probable, possible, or doubtful. The patient achieved a Naranjo score of 6, indicating a probable drug-induced reaction. This score was based on the temporal onset of symptoms following terlipressin administration, clinical improvement after discontinuation of the drug, absence of alternative causes for lactic acidosis, and previous reports documenting similar reactions. Given the clear temporal relationship between cessation of terlipressin and subsequent improvement in lactate levels alongside clinical stabilisation, terlipressin-induced vasoconstriction was deemed the most likely cause of the patient’s severe lactic acidosis (Table 2).

**Table 2:** Timeline of clinical events. NEWS: National Early Warning Score.

Time point	Clinical events					
Day 0 (Presentation)	A 58-year-old male presented with acute hematemesis (three episodes, approximately 500 mL) without melaena. Past medical history included alcohol-related liver cirrhosis and type 2 diabetes mellitus.					
Day 0 (Admission)	Haemodynamically stable on admission (blood pressure = 128/63 mmHg, heart rate = 105 bpm, NEWS = 1). Initial venous blood gas showed metabolic acidosis with elevated lactate (pH = 7.32, lactate = 8.0 mmol/L).					
Day 0	Urgent upper gastrointestinal endoscopy revealed grade III oesophageal varices; seven-band ligation was performed.					
Day 0	Terlipressin was initiated along with intravenous proton pump inhibitor, antibiotics, and tranexamic acid for the management of acute variceal bleeding.					
Within hours of terlipressin initiation	Acute clinical deterioration with drowsiness, dizziness, hypotension (blood pressure = 92/59 mmHg), and tachycardia (heart rate = 118 bpm).					
Day 0–1	Repeat venous blood gas demonstrated severe metabolic acidosis (pH = 6.97), profound hyperlactatemia (lactate = 20 mmol/L), and low bicarbonate (6 mmol/L). Blood tests showed acute kidney injury and transaminitis consistent with multi-organ dysfunction.					
Day 1	CT angiography showed patent mesenteric vessels with no arterial occlusion. Bowel wall thickening was present, suggestive of severe vasospasm.					
Day 1	Terlipressin was discontinued due to suspected drug-induced systemic vasospasm. Octreotide was initiated as an alternative vasoactive therapy.					
Day 1–2	Rapid biochemical improvement with normalisation of lactate levels and acid-base status within 24–48 hours. No further gastrointestinal bleeding occurred.					
Day 2–3	Progressive improvement in renal and liver function tests. The patient remained haemodynamically stable.					
Discharge	Patient discharged in stable condition with advice to permanently avoid terlipressin.					
Follow-up	Clinical stability was maintained with no recurrence of symptoms.					

## Discussion

Liver cirrhosis and oesophageal varices

Liver cirrhosis is a chronic liver disease characterised by fibrosis and regenerative nodules that distort normal hepatic architecture and blood flow [6]. In the United Kingdom, one study reported a prevalence of liver cirrhosis of 0.1% [7]. In 2010, liver cirrhosis accounted for approximately 2% of global deaths, corresponding to an estimated one million deaths worldwide [8].

However, oesophageal varices occur in approximately 40-95% of patients with cirrhosis, and 15-20% of these varices bleed within one to three years of diagnosis. Among survivors of variceal bleeding, approximately 30% die within two years, and around 20% experience recurrent bleeding during the same period [9,10].

Oesophageal varices are dilated submucosal veins of the oesophagus, most commonly resulting from portal hypertension due to liver cirrhosis. In addition to causing arterial vasodilation of the splanchnic circulation, portal hypertension leads to dilation of collateral vessels between the portal and systemic venous systems [11,12]. One of the main sites of these collaterals is the distal oesophagus and proximal stomach, resulting in the formation of oesophageal varices [13].

Management of acute oesophageal variceal bleeding

General management should focus on initial stabilisation and supportive care, including the following: (1) resuscitation, with prompt establishment of intravenous access and assessment of airway, breathing, and circulation. Orotracheal intubation should be considered in patients with massive haematemesis or altered mental status to protect the airway. (2) Restrictive red blood cell transfusion strategy, with transfusion recommended when haemoglobin is <7 g/dL, targeting a haemoglobin level of 7-9 g/dL. (3) Avoidance of routine correction of coagulopathy, as there is no evidence that correcting the international normalised ratio (INR) or platelet count improves outcomes in acute variceal haemorrhage. (4) Temporary discontinuation of outpatient medications, such as diuretics and non-selective beta-blockers (NSBBs), in patients with hypotension during active bleeding, as NSBBs may blunt the compensatory sympathetic response to haemorrhage.

On the other hand, specific pharmacological therapy for acute variceal haemorrhage should be initiated as soon as the diagnosis is suspected and while arranging urgent upper gastrointestinal endoscopy. This includes the following: (1) vasoactive therapy, which reduces portal pressure through splanchnic vasoconstriction. Commonly used agents include octreotide, terlipressin, and somatostatin, which have comparable efficacy: octreotide is given as an intravenous bolus of 50 μg, followed by continuous infusion at 50 μg/hour for two to five days; terlipressin is given 2 mg intravenously every four hours for the first 48 hours, followed by 1 mg every four hours for a total duration of two to five days; somatostatin is given as intravenous bolus of 250 μg, followed by continuous infusion at 250-500 μg/hour for two to five days. (2) Antibiotic prophylaxis, which reduces the risk of bacterial infections, particularly spontaneous bacterial peritonitis, and is associated with lower rates of rebleeding and mortality [14-16].

Mechanism of terlipressin and clinical use

Terlipressin exerts its therapeutic effect via V1 receptor-mediated vasoconstriction, predominantly within the splanchnic circulation. In rare cases, this vasoconstrictive effect may extend systemically, resulting in diffuse microcirculatory hypoperfusion. This leads to impaired oxygen delivery at the tissue level, a shift towards anaerobic metabolism, and rapid lactate accumulation. In patients with cirrhosis, reduced hepatic clearance of lactate may further exacerbate hyperlactatemia. The profound lactic acidosis observed in this case, in the absence of radiological evidence of mesenteric ischaemia, supports a mechanism of widespread microvascular vasospasm rather than fixed vascular occlusion [17].

Rare but severe complications

Although generally considered safe, terlipressin can, in rare instances, cause serious ischaemic complications, including myocardial ischaemia, cutaneous necrosis, peripheral ischaemia, and mesenteric ischaemia. Even more uncommon is widespread systemic vasospasm, which can precipitate severe lactic acidosis. Such presentations can closely mimic acute mesenteric ischaemia, creating diagnostic uncertainty and necessitating urgent intervention [2]. The mechanism is thought to involve excessive vasoconstriction in the microcirculation, leading to tissue hypoperfusion and impaired oxygen delivery.

Diagnostic and management challenges

Diagnosing terlipressin-induced lactic acidosis is challenging due to multiple confounding factors in patients with variceal bleeding, such as hypovolemia, haemorrhagic shock, sepsis, or liver dysfunction. In our patient, lactate levels rose sharply to 20 mmol/L within hours of terlipressin initiation, with profound metabolic acidosis (pH = 6.97). Importantly, CT angiography excluded mesenteric ischaemia, and the acidosis resolved only after discontinuation of terlipressin, supporting a diagnosis of drug-induced systemic vasospasm. The use of the Naranjo probability scale further strengthened the association, indicating a probable adverse drug reaction.

Management of this condition involves immediate cessation of terlipressin, careful haemodynamic support, and consideration of alternative vasoactive agents, such as octreotide, to maintain variceal haemostasis. Monitoring lactate trends, acid-base status, and clinical perfusion markers is essential to guide supportive care and avoid irreversible tissue injury.

Comparison with previously reported cases

Similar cases of terlipressin-induced ischaemic complications have been described in the literature, most commonly involving peripheral cyanosis, myocardial ischaemia, or mesenteric hypoperfusion. As in previously reported cases, our patient demonstrated rapid clinical and biochemical improvement following discontinuation of terlipressin. The close temporal association between drug administration, symptom onset, and recovery supports a causal relationship and is consistent with previously published case reports.

Clinical implications

This case highlights the importance of considering terlipressin-induced systemic vasospasm in patients who develop unexpected lactic acidosis after treatment initiation. Objective clinical deterioration, including hypotension, metabolic acidosis, and biochemical evidence of multi-organ dysfunction, should prompt immediate reassessment and drug cessation. Early recognition is essential, as prompt discontinuation typically leads to rapid and complete recovery.

Patient perspective

The patient expressed understanding of the clinical course and treatment provided and was appreciative of the care received during hospitalisation. The patient provided verbal consent for publication and agreed to share this clinical course to support medical learning.

Informed consent

Verbal informed consent was obtained from the patient for publication of this case report. All patient identifiers were removed, and no images revealing the patient’s identity are included.

## Conclusions

Terlipressin-induced lactic acidosis is a rare but potentially life-threatening complication that requires prompt recognition and intervention. In this case, the patient developed severe metabolic acidosis shortly after terlipressin administration for acute variceal bleeding. Clinical and biochemical recovery occurred only after discontinuation of the drug and initiation of octreotide, highlighting the importance of early identification and management to prevent irreversible tissue injury.

While terlipressin remains a cornerstone therapy for variceal haemorrhage and hepato-renal syndrome, clinicians must maintain vigilance for unexpected systemic ischaemic effects, particularly in patients with liver disease or other comorbidities. Close monitoring of lactate, acid-base status, and peripheral perfusion is essential during therapy. This case emphasises the importance of awareness of rare adverse effects, timely clinical reassessment, and the use of alternative vasoactive agents when needed, ensuring patient safety and optimal outcomes in acute variceal bleeding management.
